# Grooving and Absorption on Substrates to Reduce the Bulk Acoustic Wave for Surface Acoustic Wave Micro-Force Sensors

**DOI:** 10.3390/mi15050637

**Published:** 2024-05-09

**Authors:** Yang Feng, Haoda Yu, Wenbo Liu, Keyong Hu, Shuifa Sun, Zhen Yang, Ben Wang

**Affiliations:** 1 School of Information Science and Technology, Hangzhou Normal University, Hangzhou 311121, China; 2023112011031@stu.hznu.edu.cn (H.Y.); liuwenbo@stu.hznu.edu.cn (W.L.); hukeyong@yeah.net (K.H.); watersun@hznu.edu.cn (S.S.); 2 Mobile Health Management System Engineering Research Center of the Ministry of Education, Hangzhou 311121, China; 3 School of Engineering, Hangzhou Normal University, Hangzhou 311121, China; 4 School of Electronic Information, Huzhou College, Huzhou 313002, China; yangzhen@zjhzu.edu.cn

**Keywords:** surface acoustic wave (SAW), micro-force sensor, bulk acoustic wave (BAW)

## Abstract

Improving measurement accuracy is the core issue with surface acoustic wave (SAW) micro-force sensors. An electrode transducer can stimulate not only the SAW but also the bulk acoustic wave (BAW). A portion of the BAW can be picked up by the receiving transducer, leading to an unwanted or spurious signal. This can harm the device’s frequency response characteristics, thereby potentially reducing the precision of the micro-force sensor’s measurements. This paper examines the influence of anisotropy on wave propagation, and it also performs a phase-matching analysis between interdigital transducers (IDTs) and bulk waves. Two solutions are shown to reduce the influence of BAW for SAW micro sensors, which are arranged with acoustic absorbers at the ends of the substrate and in grooving in the piezoelectric substrate. Three different types of sensors were manufactured, and the test results showed that the sidelobes of the SAW micro-force sensor could be effectively inhibited (3.32 dB), thereby enhancing the sensitivity and performance of sensor detection. The SAW micro-force sensor manufactured using the new process was tested and the following results were obtained: the center frequency was 59.83 MHz, the fractional bandwidth was 1.33%, the range was 0–1000 mN, the linearity was 1.02%, the hysteresis was 0.59%, the repeatability was 1.11%, and the accuracy was 1.34%.

## 1. Introduction

Devices that utilize surface acoustic waves (SAWs) have found extensive applications across a range of fields, including mechanical engineering, aerospace and aviation, signal processing, non-destructive evaluation, and the realm of sensor technology, among others [1]. SAW sensors, recognized for their vital role in sensing technology, have shown great promise due to a combination of advantageous attributes. These include high sensitivity, rapid response times, excellent specificity, reversibility, battery-powered operation, small size, low cost, compact dimensions, and affordability [2]. As a result, they hold significant potential for a wide array of future applications. The SAW sensors can be used to measure temperature [3], humidity [4], gas [5,6], force [7], and so forth. 

In this paper, we focus on research into SAW micro-force sensors. It is well understood that a variety of sensor types are capable of measuring micro-forces, including but not limited to capacitive micro-force sensors, resistance micro-force sensors, fiber optic micro-force sensors, hall micro-force sensors, and so on. However, the output signals of these traditional force sensors are analog signals, which are susceptible to environmental factors, whereas the output signal of the SAW micro-force sensor is a frequency signal, which is less susceptible to interference. In addition, compared to traditional micro-force sensors, the cost of SAW force sensors is relatively low. However, due to the limitations of manufacturing traditional SAW device processes, the measurement accuracy is limited. 

For SAW micro-force sensors, the study of measuring accuracy is the core issue. To develop a practical SAW micro-force sensor, we aim to improve its measuring accuracy. To develop a high-precision SAW force sensor, the design of input and output interdigital transducers (IDTs) is critical. Xiaozhou Lü et al. [8,9] used evenly distributed fingers to design input and output IDTs, but this kind of design method can cause the input IDT to produce a second-order effect [10,11,12], which will increase the sidelobes of the SAW sensor, thus seriously affecting the measuring accuracy of it. When the input transducer excites the SAW signal, it also excites the BAW signal [13], and the BAW signal will reflect on the substrate surface and then reach the output transducer. Due to the influence of BAW on the SAW signal received by the output transducer, the device will eventually have poor sidelobe rejection performance. Therefore, it is necessary to consider and eliminate the problem of BAW in the process of realization with SAW devices.

To solve the problem of BAW in surface acoustic wave devices, many scholars have conducted some research. Changbao Wen proposed using the multistrip coupler (MSC) in SAW devices [14,15]. Wenke Lu presented the solutions to the insertion loss and the bulk wave of the wavelet transform processors using SAW devices [16]. Hua Jiang discussed the insertion loss and the sidelobe in wavelet transform and reconstruction processors using SAW devices [17]; however, the sidelobe was 28.89 dB. Therefore, regarding SAW micro-force sensors, there have been fewer studies of the second-order effect, especially the bulk acoustic waves that cause sidelobes. The bigger the sidelobe, the worse the anti-interference performance of the micro-force sensor. Therefore, we must propose the principle of BAW and methods to solve sidelobes.

In this paper, we proposed the electrode-overlap envelope to design the input IDT. We selected ST-X SiO_2_ as the material of the piezoelectric substrate to reduce the influence of sound–electricity reclamation [18]. Two methods are given to reject sidelobes: one is arranging acoustic absorbers at both ends of the substrate, and another is grooving at the bottom of the piezoelectric substrate, which can reduce the influence of BAW.

This paper is organized as follows: In Section 2, the principle of the SAW micro-force sensor is introduced. In Section 3, BAW propagation in the SAW micro-force sensor is shown. In Section 4, we discuss the manufacture of three types of SAW micro-force sensor: a SAW micro-force sensor without acoustic absorbers, a SAW micro-force sensor with acoustic absorbers, and a SAW micro-force sensor both with acoustic absorbers and grooved piezoelectric substrate. In Section 5, we measure the basic parameters of three types of SAW micro-force sensors. The conclusions are presented in Section 6.

## 2. Principle of the SAW Micro-Force Sensor

There are two kinds of SAW micro-force sensors, namely, the delay-line type and the resonator type. The delay-line structure sensor has a long acoustic transmission path, which can obtain a large sensitive area, and its structure is simple and small. Therefore, the delay-line type is adopted as the basic structure of the sensor in this paper. It is composed of a piezoelectric substrate and two IDTs, and the input IDT and output IDT are etched on a piezoelectric substrate, which is a thin and flexible diaphragm.

**Figure 1:**
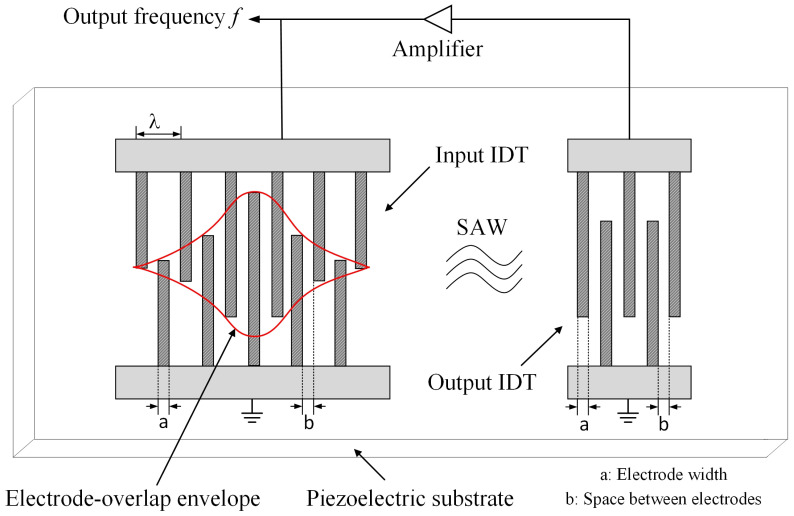
Basic structure of the SAW micro-force sensor by delay-line.

The basic structure of the SAW delay-line sensor is shown in Figure 1. Its working principle is as follows: firstly, the input electrical signal is converted into the SAW signal by the input IDT; then, the SAW signal is propagated to the output IDT through the piezoelectric medium, and finally, the SAW signal is converted into an electrical signal by the output IDT.

**Figure 2:**
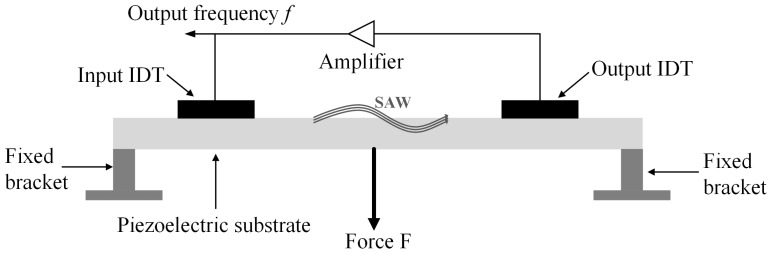
The measurement system of the SAW micro-force sensor.

The SAW micro-force sensor adopts a double-end fixed beam structure, that is, the two ends of the piezoelectric substrate are fixed, which is shown in Figure 2. When the force F is subjected to the central position of the piezoelectric substrate, its elastic stiffness constant and density will change, which will lead to variations in the propagation velocity of SAW. On the other hand, the center distance of the interdigital electrodes will change, and it will cause variations in the wavelength of it; finally, it will result in variations in the oscillation frequency of the SAW sensor. Therefore, the goal of measuring force F can be reached by the variations in the output frequency of the SAW sensor.

As shown in Figure 2, when the force F is subjected to the central position of the piezoelectric substrate, it causes variations in the piezoelectric substrate strain σ, and combined with Figure 1, we can obtain the following:

(1) $$\left\{\begin{array}{c} a\left(\sigma \right)=a_{0}\left(1+\sigma \right)\\ b\left(\sigma \right)=b_{0}\left(1+\sigma \right)\\ \;\upsilon \left(\sigma \right)=\upsilon_{0}\left(1+k\sigma \right)\\ \;\lambda \left(\sigma \right)=2a\left(\sigma \right)+2b\left(\sigma \right) \end{array}\right.$$

where $a\left(\sigma \right)$ is the width of the electrode, $b\left(\sigma \right)$ is the spacing of the electrode, $\upsilon \left(\sigma \right)$ is the propagation velocity of SAW, and $\lambda \left(\sigma \right)$ is the wavelength of SAW; in addition, $a_{0}$, $b_{0}$, $\upsilon_{0}$, and $\lambda_{0}$ are the values of $a\left(\sigma \right)$, $b\left(\sigma \right)$, $\upsilon \left(\sigma \right)$, and $\lambda \left(\sigma \right)$ when the force F = 0, respectively. The variable k denotes the elastic stiffness constant of the piezoelectric substrate. This constant embodies the non-zero pressure coefficients associated with the elastic properties, piezoelectric characteristics, and the dielectric constant of the material [19]. In addition, we know from the SAW theory that

(2) $$f=\frac{\upsilon \left(\sigma \right)}{\lambda (\sigma )}$$

where f is the output frequency of the SAW micro-force sensor, and the center frequency can be written as follows:

(3) $$f_{0}=\frac{\upsilon_{0}}{\lambda_{0}}$$

from Equations (1)–(3), we can obtain the following:

(4) $$f=\frac{\upsilon \left(\sigma \right)}{\lambda (\sigma )}=\frac{\upsilon_{0}\left(1+k\sigma \right)}{\left(2a_{0}+2b_{0})(1+\sigma \right)}=\frac{\upsilon_{0}(1+k\sigma )}{\lambda_{0}(1+\sigma )}=\frac{1+k\sigma }{1+\sigma }f_{0}$$

so the output frequency shift ${\;\Delta }_{f}$ can be written by

(5) $${\;\Delta }_{f}=f-f_{0}=\frac{(k-1)\sigma }{1+\sigma }f_{0}$$

According to the relevant mechanical theory, we can obtain the following:

(6) $$\sigma_{1}=-\frac{3FL}{4EWH^{2}}$$

where L, W, and H are the length, width, and thickness of the piezoelectric substrate, respectively, and E is the modulus elasticity of the piezoelectric substrate material. By substituting (6) into (5), we obtain the following:

(7) $${\;\Delta }_{f}=\frac{3f_{0}L(1-k)}{4EWH^{2}-3FL}F\;$$

In this paper, quartz is selected as a piezoelectric substrate with elastic modulus E = 72.7 GPa [20,21]. The magnitude of E is 10^9^ (i.e., GPa), so 4EWH >> 3FL, and then Equation (7) can be written as

(8) $$\Delta_{f}=\frac{3f_{0}L(1-k)}{4EWH^{2}}F\;$$

It can be seen from Equation (8) that there is a linear relationship between the output frequency shift ${\;\Delta }_{f}$ and the force F, so the force F can be measured by the output frequency shift ${\;\Delta }_{f}$.

## 3. The BAW Propagation in SAW Micro-Force Sensor

### 3.1. Phase Matching of IDT to BAW

Upon the application of a micro-force to the SAW sensor, deformation occurs within the piezoelectric substrate. The input IDT not only stimulates the desired surface acoustic wave signal but also stimulates the bulk acoustic wave signal that propagates through the substrate’s volume, provided that the surface projection of the wavelength aligns with the period of the IDT.

**Figure 3:**
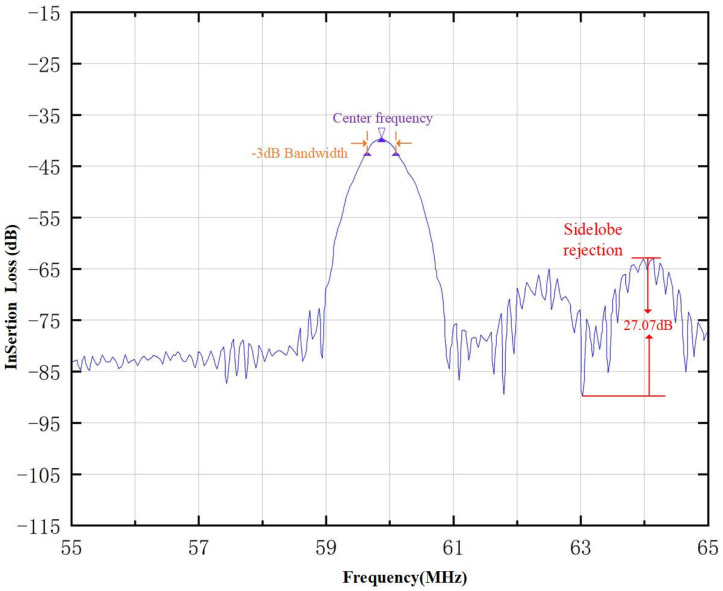
Frequency curve of SAW micro-force sensor.

The BAW signal will be reflected on the substrate surface and then reach the output transducer. Since the propagation speed of the bulk acoustic wave is larger than the surface acoustic wave, the frequency of the surface acoustic wave of the interdigital transducer cannot be greater than the frequency of the excited BAW, which is the reason why the spectrum curve above the center frequency response usually appears on the right side of the frequency test diagram of the device, as shown in Figure 3. In Figure 3, the sidelobe rejection achieves 27.07 dB.

According to Figure 1, the condition for achieving phase synchronization can be expressed as follows:

(9) $${\upsilon_{0}=f}_{0}\lambda_{0}=\frac{\upsilon \left(\gamma \right)}{cos\gamma }$$

where $f_{0}$ is the center frequency, $\lambda_{0}$ is the IDT period, $v_{0}$ is the propagation velocity, γ is the inclination angle of the bulk wave concerning the surface wave, and $\upsilon \left(\gamma \right)$ is the velocity of the wave in the γ direction.

**Figure 4:**
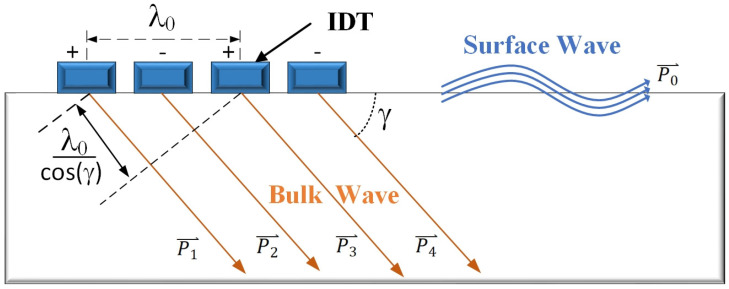
Schematic diagram of BAW stimulated by IDT.

In Figure 4, the wave vector $p_{O}$ propagating along the surface of the piezoelectric substrate is $2\pi /\lambda_{0}$. Based on the properties of wave vectors, Equation (9) can be reformulated as follows:

(10) $${\;p}_{O}=2\pi /\lambda_{0}=p_{s}=p(\gamma )cos\gamma \;$$

where $p_{s}$ is the surface component of the wave vector p(γ).

In an isotropic medium, $\upsilon \left(\gamma \right)$ depends on the wave mode, the material of the substrate, the orientation of the surface cut, the direction of the transducer, and the ambient temperature. When these factors are determined, the center frequency is determined by the inclination angle as follows:

(11) $$f_{0}=\frac{1}{\lambda_{0}}\;\cdot \;\frac{\upsilon \left(\gamma \right)}{cos\gamma }$$

where $f_{0}$ is determined by γ, specifically 1/cosγ. Therefore, the larger the propagation angle (γ) of BAW, the higher the frequency of its coupling to IDT.

When the frequency increases, one can juxtapose this scenario with the limiting condition for BAW propagation, specifically when γ is at its cutoff value $\gamma_{c}$. This condition signifies that the direction of energy transfer or wave motion is parallel to the surface. The familiar SBAW equals SSBW, propagating directly between two IDTs on the same surface [22]. By comparing the DBAW case with the limiting SBAW case, we can obtain the following from Equation (9):

(12) $$\frac{{{(f}_{0})}_{DBAW}}{{{(f}_{0})}_{SBAW}}=\frac{\upsilon \left(\gamma \right)}{v_{SBAW}}\;\cdot \frac{cos\gamma_{c}}{cos\gamma }=\frac{1}{cos\gamma }$$

The wave’s coupling efficiency to the IDT is likely to diminish as the inclination angle increases for a specific device. This reduction in coupling efficiency imposes a practical constraint on the maximum steepness of the wave that can be effectively utilized. For DBAW devices, enhancement factors (1/cosγ) of 2 or more (γ ≥ 60°) are considered achievable and reasonable under favorable material and cut conditions.

### 3.2. The BAW in ST-X Quartz Material

SAW devices are sensitive to the materials from which they are made, as the properties of the material can significantly influence the performance of the device. In a comparative study examining the application of quartz in contrast to PZT (lead zirconate titanate piezoelectric ceramics, PZT) ceramics and PMN-PT (magnesium niobate–lead titanate piezoelectric material, PMN-PT) single crystals for surface acoustic wave (SAW) devices, several key factors come into play that influence the bulk characteristics and the overall performance of the resulting SAW devices [23,24,25,26,27,28,29,30,31], as follows:

1. Nonlinear effects: PZT and PMN-PT are known for their nonlinear piezoelectric effects, which can be exploited for certain applications such as frequency multiplication or for creating devices with tunable properties. However, these nonlinearities can also introduce complexities in wave propagation that might not be present in quartz.
2. Piezoelectric saturation: At high drive levels, the piezoelectric effect in PZT and PMN-PT can saturate, leading to a nonlinear response. This can affect the accuracy and stability of the SAW device, particularly in applications that require high-power handling or large signal modulation.
3. Cutting angle: The properties of SAW devices are highly dependent on the cut and orientation of the crystal. Quartz has well-established cuts (e.g., Y-cut, AT-cut, ST-cut) that are optimized for SAW propagation.
4. Signal distortion: Nonlinear effects can cause signal distortion in SAW devices, particularly for applications that involve large signal swings or high-power operation. This can be more pronounced in PZT and PMN-PT devices compared to those made from quartz.
5. Fabrication complexity and cost: PZT and PMN-PT might be more challenging to fabricate into the precise geometries required for SAW devices, potentially leading to higher costs or lower yields.

In summary, the designing of SAW devices needs to adjust device geometries, operating frequencies, or temperature-compensation strategies to optimize performance with these alternative materials. To reduce the influence of sound–electricity reclamation on the SAW sensor, we selected ST-X quartz as the material of the piezoelectric substrate [32,33]. So, analyses should be conducted on the shear–horizontal (SH) bulk waves that travel perpendicularly to the X-axis within quartz.

**Figure 5:**
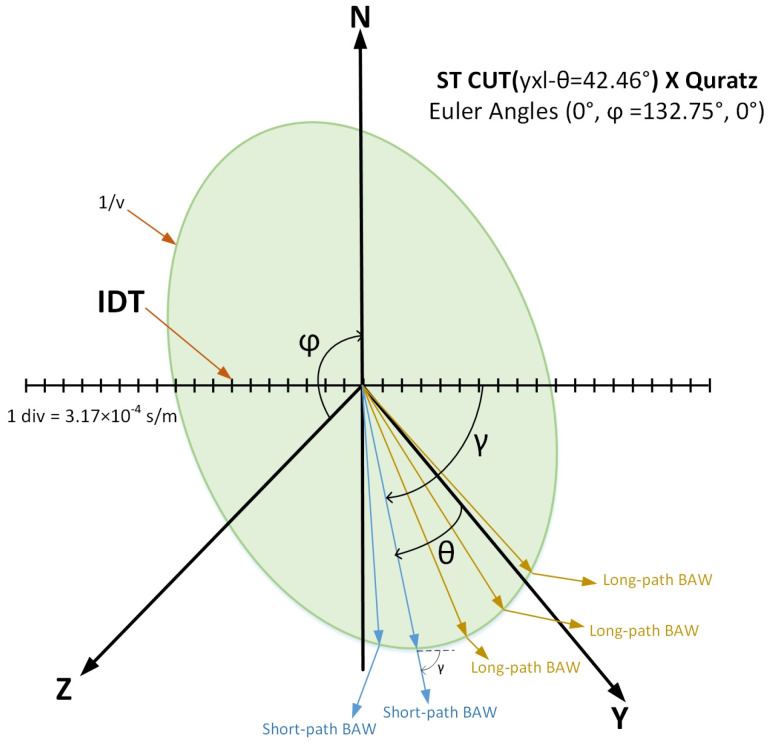
The curve for ST-cut X quartz shows the path directions of BAW.

Figure 5 illustrates the propagation of shear waves that are oriented perpendicular to the axis of two-fold symmetry, specifically the X-axis of quartz, with their polarization vector aligned parallel to this axis, resulting in X-polarized shear waves. These waves are classified as pure shear waves, which have the unique property of reflecting off surfaces that are parallel to the symmetry axis without experiencing any energy loss or mode conversion.

Additionally, Figure 5 provides a depiction of the slowness curve for a commonly used quartz substrate, known as the ST cut, along with some representative examples of the directions of long-path BAWs and short-path BAWs. A long-path BAW can stimulate the SBAW, and a short-path BAW can stimulate the DBAW. So, the solution to reduce the BAW must take these two influences into account.

## 4. Manufactured of the SAW Micro-Force Sensor

### 4.1. Arranging Acoustic Absorbers at Ends of Piezoelectric Substrate

**Figure 6:**
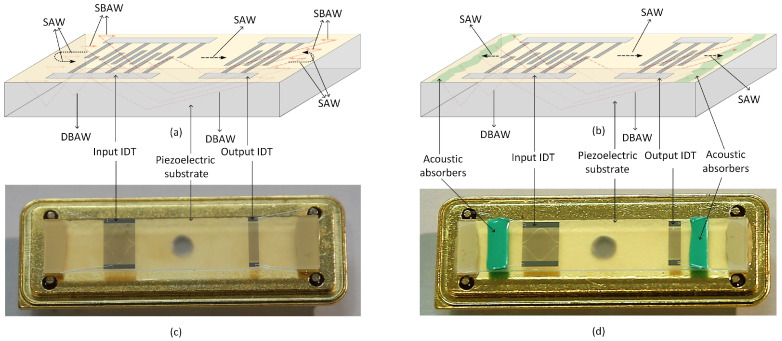
Reflection of BAW at the ends of piezoelectric substrate: (**a**) Reflection way without acoustic absorbers; (**b**) reflection way with acoustic absorbers; (**c**) physical image without acoustic absorbing glue; (**d**) physical image with acoustic absorbers.

When the acoustic wave propagates on the surface and the body of the piezoelectric substrate, the sudden fracture of the edge of the substrate will cause the surface acoustic wave to reflect the input IDT and output IDT, forming a false acoustic wave reflection, and eventually increasing the ripple in the frequency response of the surface acoustic wave device, as shown in Figure 6. This phenomenon is called the transducer edge reflection phenomenon.

When the acoustic absorbers are seated and glued to the edge of the piezoelectric substrate, the absorbing material can better absorb the reflection of the pseudo surface wave, thus solving the problem of the edge reflection of the transducer, as shown in Figure 6. Commonly used acoustic-absorbing substances are silicone rubber and other substances.

### 4.2. Piezoelectric Substrate with Slotted Bi-Directional Grooves

The slotting process involves utilizing a specialized tool known as a slice cutter to meticulously create a narrow groove in a designated direction on the rear surface of the piezoelectric substrate. This action effectively impedes the propagation of BAW signals along their typical transmission path [34,35].

**Figure 7:**
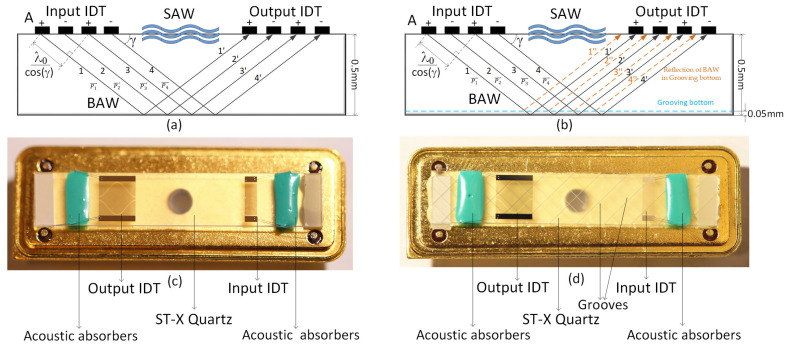
Change in BAW reflection excited by IDT after the grooves. (**a**) Reflection of BAW without grooved piezoelectric substrate; (**b**) BAW reflection with grooved piezoelectric substrate; (**c**) physical image of ungrooved sensor; (**d**) physical image of grooved sensor.

In Figure 7a, the path labeled as 1 represents the route taken by electrode A of the input IDT, while the path labeled as 1′ indicates the reflection of the BAW that occurs before the implementation of the slotting process. In Figure 7b, the path marked with 1′’ (depicted by an orange dotted line) signifies the reflection of the BAW that takes place after the grooving process has been completed. This slotting process results in a modification of the piezoelectric substrate’s thickness, specifically a reduction from 0.5 mm to 0.45 mm due to a slot depth of 0.05 mm. This alteration repositions the point of maximum BAW reflection to the termination of the central area’s output IDT, which substantially diminishes the impact of the BAW. Figure 7c shows the physical image of the ungrooved sensor. Figure 7d shows the physical image of the grooved sensor.

**Figure 8:**
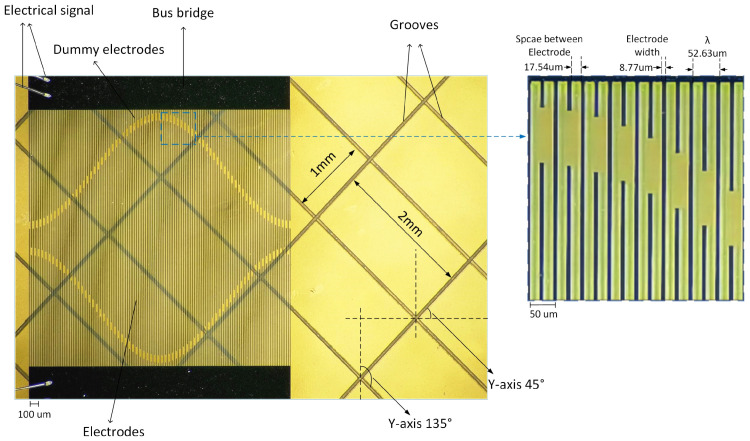
Grooves on the piezoelectric substrate of SAW micro-force sensor (magnified view with 120×).

Figure 8 provides a magnified view of the SAW micro-force sensor, with a magnification factor of 120×. In Figure 8, the grooves are slotted at the bottom of the piezoelectric substrate to avoid the BAW reflection arriving at the central area output IDT. The pattern of the grooves is set at intervals along the Y-axis: a 2 mm gap in the direction of 45 degrees and a 1 mm gap in the direction of 135 degrees.

## 5. Measurement of the SAW Micro-Force Sensor

Three kinds of SAW micro-force sensors were fabricated on ST-X quartz substrate: the SAW micro-force sensor without acoustic absorbers, the SAW micro-force sensor with acoustic absorbers, and the SAW micro-force sensor both with acoustic absorbers and grooved piezoelectric substrate. In addition, the electrode material of the IDTs was aluminum [36], and the electrode-overlap envelope of the input IDT was weighted according to the Hamming function [37,38,39].

Table 1 shows the design parameters of the micro-force sensor fabricated on ST-X quartz. We manufactured three types of sensors according to the parameters in Table 1.

**Figure 9:**
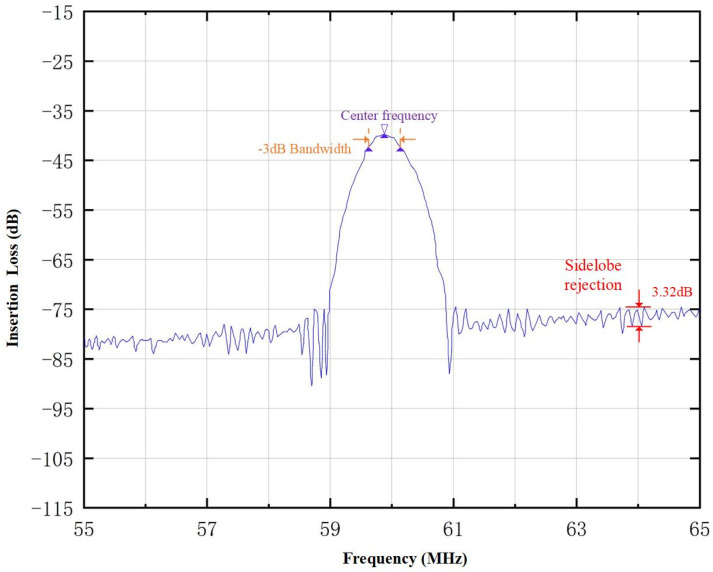
Frequency curve of SAW micro-force with acoustic absorbers and grooved piezoelectric substrate.

Figure 9 shows the frequency curve of the SAW micro-force with acoustic absorption and grooved piezoelectric substrate. When the grooves are engraved on the piezoelectric substrate of the SAW micro-force tension sensor, the sidelobe (3.32 dB) can be effectively suppressed. Compared with Figure 3, we can see clearly that with the acoustic absorption and the grooved piezoelectric substrate, the sidelobe of frequency characteristics is reduced from 27.07 dB to 3.32 dB, so the frequency characteristic of the SAW yarn tension sensor is better. Therefore, the method of acoustic absorption and grooved piezoelectric substrate can solve the problem of sidelobe rejection.

We loaded this type of SAW micro-force sensor and obtained the measurement data, as shown in Table 2. Table 2 shows the test of the micro-force sensor from 0 mN to 1000 mN, and each set of data is the average of 10 measurements. And we tested the basic parameters of the SAW micro-force sensor, as shown in Table 3.

## 6. Conclusions

In this paper, a geometric model has been established to describe the propagation of BAW generated by IDTs, and its fundamental characteristics have been validated through experimental means. The study takes into account the anisotropy effects of wave propagation and calculates the phase matching between the IDT and the bulk waves. Two solutions are shown to reduce the influence of BAWs for SAW micro sensors. First, the acoustic absorbers at the ends of the substrate absorb the BAW reflection. Second, grooving is at the bottom of the piezoelectric substrate to avoid the BAW reflection arriving at the central-area output IDT.

BAW configuration was carried out, and the BAW mode and material were selected. Most of the BAWs were scattered due to the acoustic absorbers and engraved grooves on piezoelectric substrates, which suppressed the sidelobe from 27.07 dB to 3.32 dB, achieving the high sidelobe rejection to the SAW micro-force sensor. We developed a new SAW micro-force sensor and tested it; the experimental results show that the center frequency was 59.83 MHz, the fractional bandwidth was 1.33%, the range was 0 mN–1000 mN, the linearity was 1.02%, the hysteresis was 0.59%, the repeatability was 1.11%, and the accuracy was 1.34%.

## Figures and Tables

**Table 1:** Design parameters of the micro-force sensor fabricated on ST-X quartz.

Piezoelectric Substrate			Center Frequency (MHz)	−3 dB Bandwidth (MHz)	Electrode Width (μm)	Space between Electrodes (μm)	Electrode Number	
Length (mm)	Width (mm)	Thickness (mm)					Input IDT	Output IDT
28	6	0.5	59.83	0.798	8.77	14.54	73	25

**Table 2:** Measurement data of SAW micro-force sensor from 0 mN to 1000 mN.

F(mN) Δf(Hz)	20 284	40 571	60 858	80 1139	100 1441	140 1993	160 2278	180 2564	200 2872
F(mN) Δf(Hz)	220 3129	240 3417	260 3688	280 3969	300 4167	340 4835	360 5117	380 5402	400 5652
F(mN) Δf(Hz)	420 5953	440 6257	460 6541	480 6801	500 7034	540 7660	560 7948	580 8256	600 8427
F(mN) Δf(Hz)	620 8799	640 9066	660 9357	680 9662	700 9907	740 10,496	760 10,786	780 11,025	800 11,328
F(mN) Δf(Hz)	820 11,641	840 11,932	860 12,219	880 12,507	900 12,855	940 13,359	960 13,641	980 13,923	1000 14,227

**Table 3:** Basic parameters of the SAW micro-force sensor.

Range	Linearity	Hysteresis	Repeatability	Accuracy
0–1 N	1.02%	0.59%	1.11%	1.34%

## Data Availability

The original contributions presented in the study are included in the article, further inquiries can be directed to the corresponding authors.
